# DNA barcoding and taxonomy: dark taxa and dark texts

**DOI:** 10.1098/rstb.2015.0334

**Published:** 2016-09-05

**Authors:** Roderic D. M. Page

**Affiliations:** Institute of Biodiversity, Animal Health and Comparative Medicine, College of Medical, Veterinary and Life Sciences, University of Glasgow, Glasgow G12 8QQ, UK

**Keywords:** DNA barcoding, taxonomy, dark taxa, dark texts, digitization

## Abstract

Both classical taxonomy and DNA barcoding are engaged in the task of digitizing the living world. Much of the taxonomic literature remains undigitized. The rise of open access publishing this century and the freeing of older literature from the shackles of copyright have greatly increased the online availability of taxonomic descriptions, but much of the literature of the mid- to late-twentieth century remains offline (‘dark texts’). DNA barcoding is generating a wealth of computable data that in many ways are much easier to work with than classical taxonomic descriptions, but many of the sequences are not identified to species level. These ‘dark taxa’ hamper the classical method of integrating biodiversity data, using shared taxonomic names. Voucher specimens are a potential common currency of both the taxonomic literature and sequence databases, and could be used to help link names, literature and sequences. An obstacle to this approach is the lack of stable, resolvable specimen identifiers. The paper concludes with an appeal for a global ‘digital dashboard’ to assess the extent to which biodiversity data are available online.

This article is part of the themed issue ‘From DNA barcodes to biomes’.

## 1. Introduction

As with many fields, digitization is having huge impact on the study of biodiversity. Museums and herbaria are engaged with turning physical, analogue specimens into digital objects, whether these are strings of As, Gs, Cs and Ts from DNA sequencing machines, or pixels obtained from a digital camera. Libraries and commercial publishers are converting physical books and articles into images, which are then converted into strings of letters using optical character recognition (OCR). Despite, sometimes, the acrimonious relationship between morphological and molecular taxonomy, there are striking parallels between the formation of DNA sequence databases in the twentieth century and the rise of natural history museums in the preceding centuries [1,2].

Viewed in this way, both classical taxonomy and genomics are in the business of digitizing life. Some of the challenges faced are similar, for example, algorithms developed for pairwise sequence alignment have applications in extracting articles from OCR text [3]. However, in other respects, the two fields are very different. Sequence data are approximately doubling every 18 months [4], whereas the number of new taxa described each year has remained essentially constant since the 1980s (see below). A challenge for sequence databases is how to handle exponential growth of data; for taxonomy, the challenge is often how to make a dent in the vast number of objects that do not have a digital representation [5]. This paper explores some of these issues, focusing on taxonomy and DNA barcoding.

## 2. Taxonomy

Among the many challenges faced by taxonomy is the difficulty of determining the size of the task it faces. Estimates of the number of species on Earth are uncertain and inconsistent, and show no signs of converging [6]. Some estimates, based on models of taxonomic effort, suggest that two-thirds of all species have already been described [7]. Analyses that use the number of authors per species description as a proxy for effort [8] ignore the global trend for an increasing number of authors per paper [9] and assume that the effort required per species description has remained constant over time. An alternative interpretation is that the quality of taxonomic description is increasing over time [10], reflecting both increased thoroughness and the availability of new technologies [11,12].

**Figure 1.**
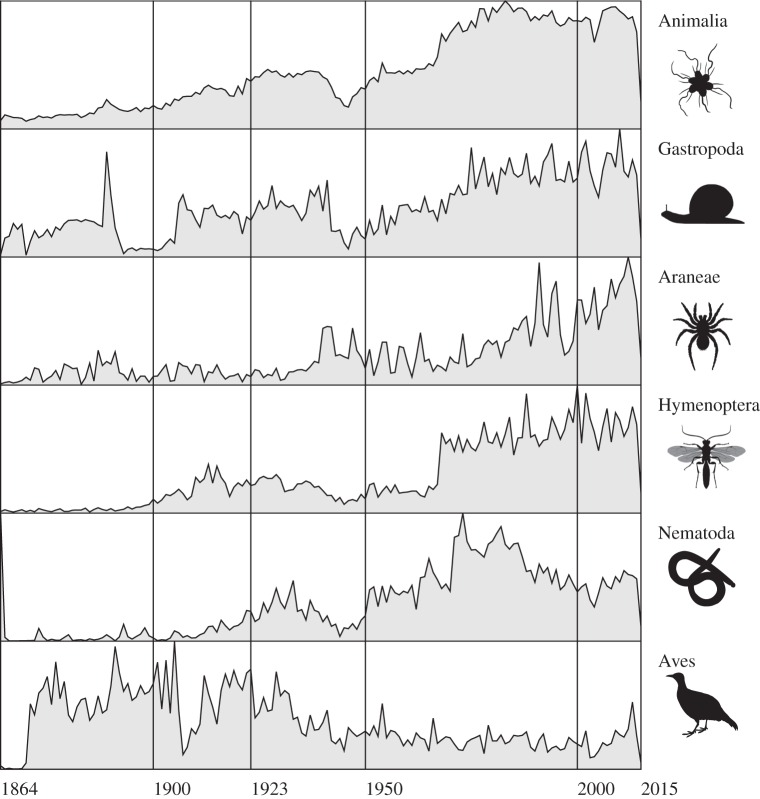
Trends of numbers of new names published each year for animals as a whole, and various taxonomic groups based on data in *Index to organism names* (ION) and BioNames (1923 is the year most published works became out of copyright in the USA). Animal pictures are from http://phylopic.org, and are either in the public domain or available under a Creative Commons CC-BY licence (Hymenoptera by Melissa Broussard; Nematoda by Michelle Site).

Rather than try and estimate an unknown (the number of species remaining to be described), here I focus on the current state of taxonomic knowledge. Given that we lack a comprehensive, global index of all species descriptions, discovering what we know about what we know is not entirely straightforward. For zoology, the nearest we have is the *Index to organism names* (ION, http://www.organismnames.com), which is based on *Zoological Record*. Figure 1 shows the numbers of new taxonomic names covered by the International Code on Zoological Nomenclature (animals plus some protozoan groups) that have been described each year based on data from ION, cleaned and augmented in BioNames (http://bionames.org) [13]. These data show an increase in overall numbers over time, with dips around the times of the two World Wars, followed by an essentially constant number each year since the mid-twentieth century. The pattern varies across taxa; some taxa show increasing numbers per year, but other taxonomic groups are essentially static or in decline, even in groups thought to be hyperdiverse such as nematodes [14].

### (a) Digitizing the taxonomic literature

The rate of progress in biodiversity research is controlled by two factors, the speed with which we can discover and describe biodiversity, and the speed with which we can communicate that information [15]. Unlike most biological disciplines, the entire corpus of taxonomic literature since the mid-eighteenth century remains a vital resource for current-day research. In this way, taxonomy is similar to the digital humanities, which has not just ‘big data’ but ‘long data’ [16]. Not only is this because of the rules of nomenclature, which dictate (with some exceptions) that the name to use for a species is the oldest one published, it also reflects the uneven effort devoted to the study of different taxonomic groups [17]. For poorly known groups, the bulk of our knowledge of their biology may reside in the primary taxonomic literature.

Digitization is one step towards making taxonomic information available. Many commercial publishers have, on the face of it, done the taxonomic community a great service by digitizing whole back catalogues of relatively obscure journals. However, digitization is not the same as access, and many commercial publishers keep this scanned literature behind paywalls. In some fields, legal issues around access have been side-stepped by constructing a ‘shadow’ dataset that summarizes key features of the data while still restricting access to the data itself. For example, by extracting phrases comprising a set of *n* words (*n*-grams) from Google Books, it is possible to create a dataset that contains valuable information without exposing the full text [18]. However, for taxonomic work, there does not seem to be an obvious way to extract a shadow. Agosti and co-workers [19,20] have explored ways to extract core facts from the literature and re-purpose these without violating copyright, though how many of their conclusions can be generalized across different national and international legal systems remains untested.

Apart from commercial digitization of the scientific literature, two other developments are accelerating access to taxonomic information. The first is the rise of open access publishing, notably journals such as *ZooKeys* that support sophisticated markup of the text [21]. This is increasing the number of recently described species that are published in a machine-readable form that can then be subject to further processing [22]. At the same time, the Biodiversity Heritage Library (BHL; http://biodiversitylibrary.org) [23] has embarked on large-scale digitization of legacy taxonomic literature. Although initially focusing on out of copyright literature (i.e. pre-1923 in the USA), BHL is increasingly getting permission from copyright holders to scan more recent literature as well. Coupled with tools such as BioStor (http://biostor.org) to locate and extract articles within the scanned volumes, BHL is fast becoming the largest available open access archive of biodiversity literature.

**Figure 2.**
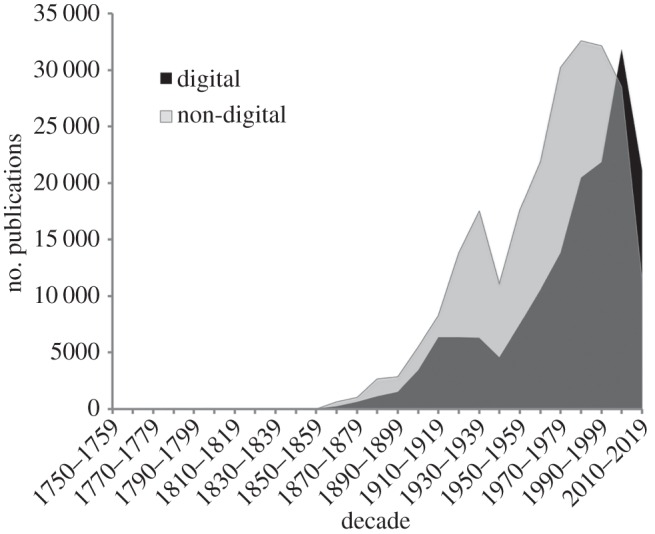
Number of taxonomic publications in BioNames for each decade, grouped by whether the publication has a digital identifier (e.g. a DOI, a link to JSTOR, BHL, BioStor, etc.). Publications containing new taxonomic names but lacking a digital identifier outnumber those that do have an identifier until 2000, represented here by the non-digital publication distribution (light grey) obscuring the digital distribution (black) until that date. The decline in both categories at the right of the chart reflects incomplete data for the current decade (2010–2020).

To quantify the extent to which the taxonomic literature has been digitized, for each decade, I counted the number of publications of new names in animals both with and without a digital identifier (such as a DOI, a PDF, a Handle or a URL to BioStor) in BioNames. The recent taxonomic literature is mostly digital: for the years 2010–2015, 60% of publications have a digital identifier, the bulk of these having a DOI. However, prior to the twenty-first century, more publications lack identifiers than have them, with the 1970s being the least digitized decade (figure 2).

### (b) The long tail of taxonomic literature

**Figure 3.**
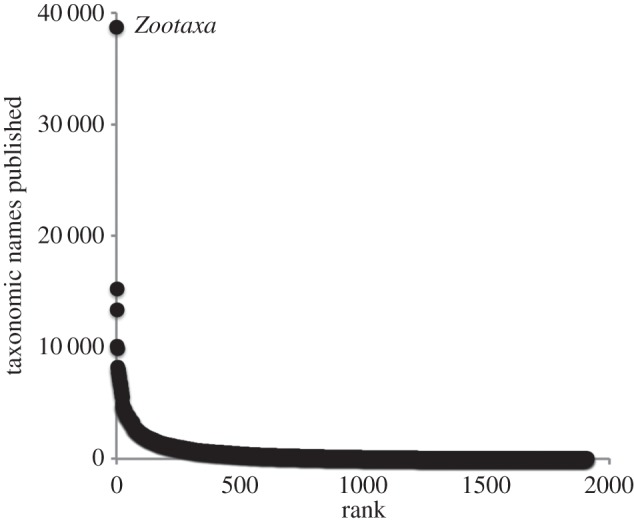
Number of taxonomic names published in each journal plotted against rank order for that journal. Note the distinctiveness of the first-ranked journal (*Zootaxa*). Data from BioNames.

Another challenge presented by the taxonomic literature is that it is highly decentralized, being spread across numerous journals (figure 3). What is striking is the dominance of animal taxonomy by the ‘megajournal’ *Zootaxa*, and yet this journal has published only 15% of the new names that have been minted since 2000. The taxonomic literature has a very ‘long tail’ of small, often obscure journals that contain a few taxonomic publications. Long tails require significant effort to index [24] although the *Zoological Record* claims 90% coverage of the taxonomic literature [25], in some taxa, there may be significantly greater gaps [26]. Conversely, if we set our sights lower, then long tail distributions mean that we can get a substantial fraction of the names from a small number of journals (the ‘low hanging fruit’). Indeed, the first 20% of the journals in figure 3 contain 80% of the names in BioNames that are linked to a publication. Unfortunately, many of these journals are not currently available digitally.

The picture that emerges from our knowledge of the taxonomic literature is the recent literature is mostly digital, identified with DOIs, and some of it is open access. However, much of our fundamental knowledge of the world's biodiversity, particularly that published in the mid-to-late-twentieth century remains digitally inaccessible (figure 2). Between the twenty-first century trend towards digitization and open access and the removal of restrictions pre-1923 as copyright expires lies a great body of twentieth century work that will require considerable effort to make available.

### (c) Genomics

In contrast with taxonomic knowledge, which is widely scattered, most genomic information is highly centralized, being stored in the three components of the International Nucleotide Sequence Database Collaboration (INSDC), namely GenBank, EMBL and the DDBJ [27]. Taxonomic name ‘databases’ more closely resemble digitized library catalogues, whereas sequence databases contain the actual sequences, which means we can compute over them. For example, a researcher with a new sequence can discover a lot about that sequence by a simple BLAST search [28], whereas a taxonomist armed only with a name will struggle to get computable data from the name alone.

Although the bulk of the world's sequence data are available in the INSDC, this is not the case for DNA barcodes, most of which reside in the Barcode of Life Data system (BOLD) [29]. Since 2009, BOLD has released some 2.5 million DNA barcodes, with updates every few months. Discovering how many of these barcodes are in GenBank is not entirely straightforward. Barcodes in GenBank may be flagged with the ‘BARCODE’ keyword (531 469 sequences at the time of writing), have a ‘LinkOut’ pointing to the BOLD database (60 684 sequences), or be listed the BioProject database [30] under accession PRJNA37833 (194 727 sequences). Because an individual sequence may meet one or more of these criteria, the sum total of sequences found by these searches (786 880) overestimates the total number of barcodes found by these methods. However, there are many barcode sequences that do not match any of these criteria. A dataset supplied by Sujeevan Ratnasingham lists 2 645 177 publicly available DNA barcodes in BOLD of which only half (1 317 132) have been shared with GenBank. The other half remain ‘siloed’ in BOLD.

### (d) Dark taxa

**Figure 4.**
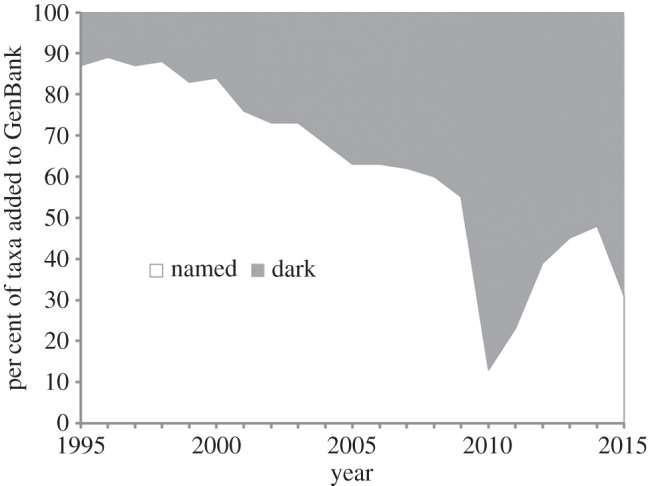
Growth of dark taxa in GenBank for invertebrate sequences. For each year, the graph shows the percentage of species-level ‘invertebrate’ taxa added during that year that do not have formal scientific names. The prominent drop in relative proportion of named taxa around 2010 is due to the addition of DNA barcodes from BOLD that lacked formal scientific names.

As desirable as data sharing is, it is not without complications. In 2011, I coined the phrase ‘dark taxa’ (http://iphylo.blogspot.co.uk/2011/04/dark-taxa-genbank-in-post-taxonomic.html; see also [31]) to refer to species in GenBank that lacked formal scientific names. Typically, they will have a name that comprises a genus name and some combination of letters and numbers to make the name unique within GenBank (e.g. a specimen code or the first letter of the last names of the researchers that deposited the sequence). For this paper, I have updated the analysis to include sequences published up to the time of writing (figure 4).

The pattern shown in figure 4 likely reflects a combination of processes. If most of the taxa being added to GenBank represent species that have already been described, then the rate at which taxa can be identified (either by taxonomists or by researchers using their outputs, such as keys) is being outstripped by the pace of sequencing. Alternatively, dark taxa may represent unknown species, but we lack taxonomists capable of recognizing the taxa as new (and formally describing them). If taxonomic capacity is a limiting factor, then we would expect a gradual decline in percentage of named taxa, which is the background pattern in figure 4. The growth of dark taxa might also reflect changing practices of molecular workers, for example in DNA barcoding where large numbers of specimens are sequenced and deposited into GenBank labelled with specimen codes rather than taxonomic names. Indeed, the dramatic increase in the numbers of dark taxa in 2010 is mostly due to sequences from the BOLD project (recognized by taxa with the prefix ‘BOLD’) being added. Even if we allow for the import of unidentified BOLD sequences as a one-off event, at present less than half the newly sequenced invertebrate taxa being added to GenBank have been identified to species level. We have little idea whether these dark taxa represent newly discovered biodiversity, or are taxa that we already know about but have simply failed to link to already described species.

### (e) Integrating biodiversity information

**Figure 5.**
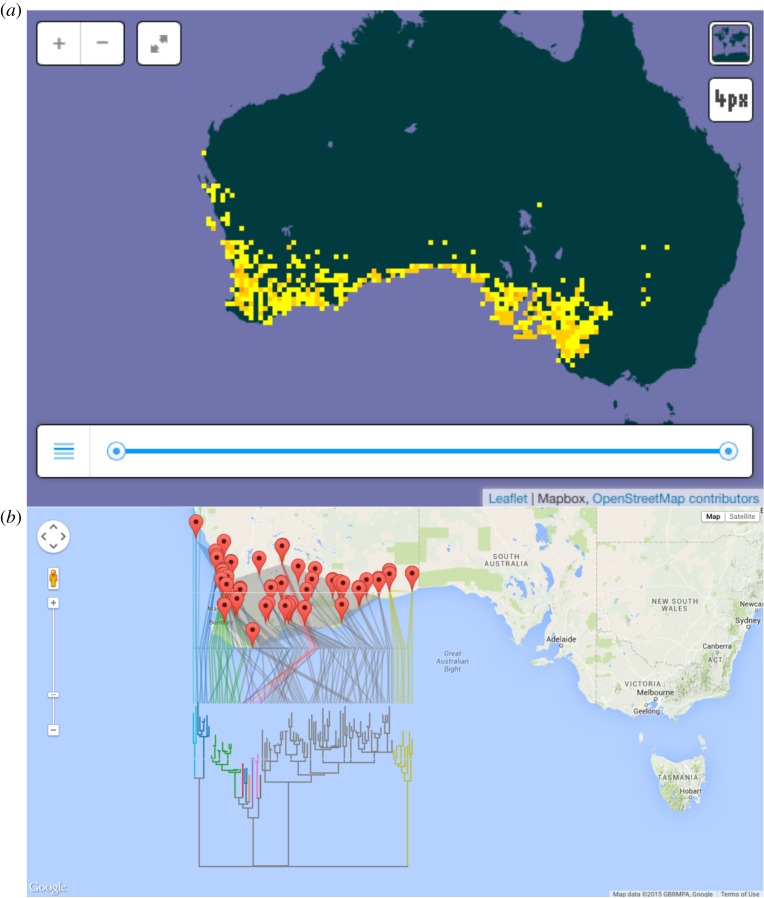
Comparison of *Morethia obscura* in GBIF (*a*) with DNA barcodes from the same taxon in BOLD (*b*). Note that the phylogeographic structure in the sequence data (which are assigned to several different BINs) implies the existence of multiple species within *Morethia obscura*.

Typically, integration across biodiversity databases is achieved using taxonomic names [32], but the rise of dark taxa makes this problematic for an increasing fraction of sequence-based data. Even if we have names, these need not always mean the same thing [33]. As an example, figure 5*a* shows the distribution of the lizard *Morethia obscura* from the Global Biodiversity Information Facility (GBIF). For comparison, figure 5*b* shows a geophylogeny [34] for some DNA barcodes from BOLD for *Morethia obscura*, which reveals considerable phylogenetic structure within ‘*Morethia obscura*’. Specimens of this species are assigned several distinct Barcode Index Numbers (BINs) [35], implying that ‘*Morethia obscura*’ comprises more than one species.

Although GBIF and BOLD present rather different views of the ‘same’ species, there is considerable overlap in the specimens used to construct figure 5*a,b*. For example, DNA barcode WAMMS012-10 was obtained from specimen WAMR127637, which also occurs in GBIF (as occurrence 691832269). Because the taxonomic concepts in GBIF and BOLD are explicitly defined with respect to sets of specimens, we can directly compare them, rather than rely on the possibly erroneous assumption that a given taxonomic name means the same thing in the two databases. Furthermore, as increasing numbers of type specimens are sequenced [36], we can more firmly associate names with sets of specimens, leading to a computable nomenclature where the name we assign to a set of specimens can be determined automatically [37]. Hence, our databases could be a lot more robust to the continual name changes that result from a nomenclatural system whereby taxonomic names are not ‘opaque identifiers’ but instead convey information about relationships (e.g. species sharing the same genus name are interpreted as being more closely related than those that do not).

**Figure 6.**
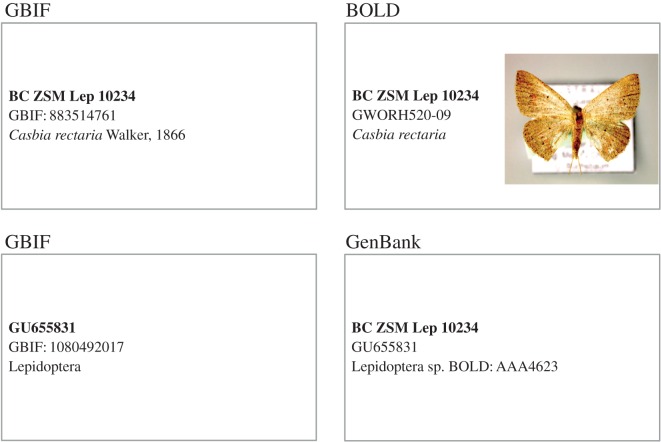
Illustration of multiple records for the same specimen of *Casbia rectaria* in GBIF. The voucher specimen for the DNA barcode is in GBIF (provided by the institution housing the specimen). The COI barcode sequence from this specimen is in both BOLD and GenBank, and because the sequence is georeferenced it is in GBIF as part of a dataset of georeferenced DNA sequences.

Integrating databases using specimens is attractive, but not without its own set of problems. The biodiversity informatics community has yet to standardize identifiers for specimens, despite numerous efforts [38,39]; consequently, there may be little apparent overlap between specimen identifiers in different databases [40]. As an example, despite the limited sharing of data between BOLD and GBIF, there are already barcoded specimens in GBIF. To illustrate, consider the DNA barcode GWORH520-09 from sample ‘BC ZSM Lep 10234’. GBIF does not have this record from BOLD, but it does have the specimen BC ZSM Lep 10234 provided by the host institution [41]. The DNA barcode from this specimen is also in GenBank, and because that record is georeferenced, it has been ingested by GBIF as part of the geographically tagged INSDC sequences dataset [42]. Hence, GBIF has duplicate records for this barcoded moth, neither provided directly by BOLD (figure 6). Merging and de-duplicating specimen-based records is going to be a significant challenge for global aggregators such as GBIF.

## 3. Summary

Both taxonomy and barcoding are actively digitizing the living world. The description of new animal taxa is essentially proceeding at a constant rate, generating a steadily growing legacy of taxonomic literature into which digitization has made modest inroads. In contrast, nucleotide sequence databases are growing exponentially. Nucleotide sequences are ‘born digital’ and readily computable; for example they can be clustered into BINs of similar sequences, or phylogenies of the type shown in figure 5. Given the obvious overlap between the goals of classical taxonomy and barcodes, the lack of digital overlap between these two endeavours is disconcerting. Many barcodes lack taxonomic names (‘dark taxa’), and much of the primary taxonomic literature has not been digitized (‘dark texts’). Integrating barcodes and taxonomy at scale is going to be significant challenge, as indeed will be integrating barcodes into mainstream sequence databases. Mapping between databases using taxonomic names seems the obvious approach, but the abundance of dark taxa shows this has not been entirely successful. Alternatives such as integration via specimens show promise, but are hampered by the lack of stable specimen identifiers. If we are to make progress the stubborn problem of the lack of unique, persistent identifiers, and crosslinks between those identifiers needs to be tackled in earnest [43,44].

As a postscript, in writing this opinion piece, I have had to write custom scripts to query various databases in an *ad hoc* manner (see http://github.com/rdmpage/dna-barcode-paper), trying to extract and assemble information that gives insight into the current state of biodiversity digitization. For these analyses and visualizations to have broader utility, it would be desirable to have some way of consistently and automatically doing these analyses, in effect creating a ‘dashboard’ of digitization that would enable us to not only see where we are as a field, but also suggest directions in which we could be heading. Many of the projects discussed in this article (mine included) use tools such as Google Analytics to provide detailed data on how users interact with their web sites [45]; it would be desirable to have similarly sophisticated tools to explore the actual data those sites are providing.

## Data accessibility

Data and scripts used to create the figures are available from GitHub repository for this article at https://github.com/rdmpage/dna-barcode-paper.
